# Dynamic assembly of ribbon synapses and circuit maintenance in a vertebrate sensory system

**DOI:** 10.1038/s41467-019-10123-1

**Published:** 2019-05-15

**Authors:** Haruhisa Okawa, Wan-Qing Yu, Ulf Matti, Karin Schwarz, Benjamin Odermatt, Haining Zhong, Yoshihiko Tsukamoto, Leon Lagnado, Fred Rieke, Frank Schmitz, Rachel O. L. Wong

**Affiliations:** 1 Department of Biological Structure, University of Washington, Seattle, 98195 WA USA; 2 Department of Neuroanatomy, Medical School Homburg/Saar, Institute for Anatomy and Cell Biology, Saarland University, Homburg/Saar, 66421 Germany; 3 Institute of Anatomy, University of Bonn, Bonn, 53115 Germany; 4 Vollum institute, Oregon Health and Science University, Portland, 97239 OR USA; 5 Department of Biology, Hyogo College of Medicine, Nishinomiya, 663-8501 Hyogo Japan; 6 School of Life Sciences, University of Sussex, Brighton, BN1 9QG UK; 7 Department of Physiology and Biophysics, University of Washington, Seattle, 98195 WA USA

**Keywords:** Development of the nervous system, Neural circuits, Visual system

## Abstract

Ribbon synapses transmit information in sensory systems, but their development is not well understood. To test the hypothesis that ribbon assembly stabilizes nascent synapses, we performed simultaneous time-lapse imaging of fluorescently-tagged ribbons in retinal cone bipolar cells (BCs) and postsynaptic densities (PSD95-FP) of retinal ganglion cells (RGCs). Ribbons and PSD95-FP clusters were more stable when these components colocalized at synapses. However, synapse density on ON-alpha RGCs was unchanged in mice lacking ribbons (*ribeye* knockout). Wildtype BCs make both ribbon-containing and ribbon-free synapses with these GCs even at maturity. Ribbon assembly and cone BC-RGC synapse maintenance are thus regulated independently. Despite the absence of synaptic ribbons, RGCs continued to respond robustly to light stimuli, although quantitative examination of the responses revealed reduced frequency and contrast sensitivity.

## Abstract (web summary)

Ribbon synapses in our sensory nervous system are central to hearing and sight, yet little is known about how these synapses are assembled and maintained during development. In this study, authors use live imaging techniques to monitor ribbon appearance, loss and maintenance in a retinal circuit during development to show that nascent synapses comprising of both ribbons and PSD95 are more stable over time compared to contacts without ribbons.

## Introduction

Synapses of many types of sensory neurons contain a specialized ‘ribbon-like’ presynaptic structure^1–3^. Vesicles containing the neurotransmitter glutamate are found tethered to the ribbons, which are anchored at active zones where transmitter release occurs^1,3^. Vesicles at the base of the ribbon are poised for fast transmitter release, ideal for reporting the rapid onset of stimuli, whereas those further up the ribbon contribute to continuous or sustained transmission, well suited for signaling the intensity and duration of a stimulus^1–4^. Ribbon synapses are characteristic of primary sensory neurons such as the light-sensitive photoreceptors in the vertebrate retina and the pineal organ and mechanosensitive hair cells in the cochlea, the vestibular organ and the fish lateral line^1–4^. Although we have gained significant knowledge of the physiological properties and structural arrangements of ribbon synapses over the years, less is known about how these synapses are assembled and maintained during development.

Previous studies suggest that the formation of presynaptic ribbons does not necessarily rely on contact and differentiation of the postsynaptic processes. In the cochlea, a presynaptic complex containing ribbons forms in hair cell terminals before postsynaptic densities are apparent^2,5^. Similarly, ultrastructural studies suggest that ribbons are present in retinal photoreceptor axon terminals before postsynaptic horizontal cell processes invaginate the terminal, and prior to the elaboration of dendrites from their other postsynaptic partner, the bipolar cells (BCs)^6–8^. Ribbons are present in hair cells of the zebrafish lateral line in the absence of afferent fibers of the lateral line ganglion neurons, although often these structures are displaced from the active zone^9^. Such misplacements have also been found in hair cells isolated in culture^5^ or when afferent fibers are damaged^10^. In addition, knockdown of *ribeye* in zebrafish, which leads to a decrease in ribbon numbers in the hair cells, results in a reduction of afferent contact and perturbed postsynaptic density distribution^11^. Collectively, these findings suggest that ribbons may form prior to postsynaptic specializations and may play instructive roles in postsynaptic differentiation at synapses made by primary sensory neurons.

**Fig. 1:**
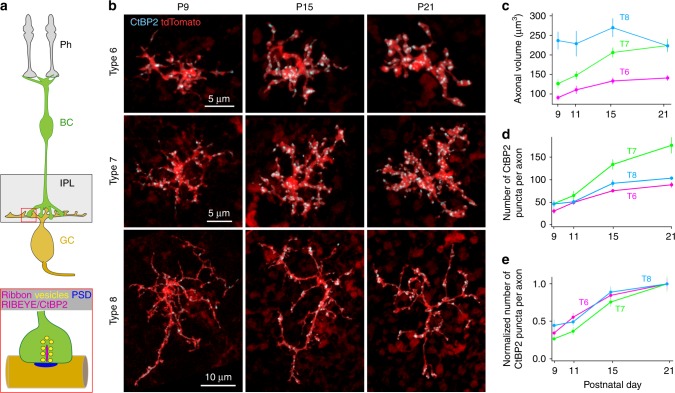
Number of axonal ribbons of distinct ON BC types across development. **a** Schematics showing the key neurons of this study and their ribbon synapses. Ph photoreceptors, BC bipolar cell, IPL inner plexiform layer, GC ganglion cell, PSD postsynaptic density. **b** Confocal images showing individual axon terminals and ribbons of three different types of ON BCs across development. Ribbons were labelled by anti-CtBP2 and those outside the axon terminals were excluded digitally (see Methods for details). **c** Axonal arbor volume of the three BC types. **d** Developmental increase in the total number of ribbons per axon, and **e** the fraction of ribbons (CtBP2 positive puncta) normalized to the average total ribbon number per axon at P21, for each BC type studied. Data were collected from 6–10 cells from 4–8 retinas for each age-group. Data are presented as mean ± sem

The assembly of ribbon synapses beyond those of primary sensory neurons (e.g., hair cells, photoreceptors) and the requirement of ribbons for synapse maintenance are, however, not well explored. To fill these gaps in knowledge, we focused on circuitry in the inner retina of vertebrates, where ribbon synapses play a key role in the transmission of information from the outer retina to the retinal output neurons, the retinal ganglion cells (RGCs). In the inner plexiform layer (IPL) of the mouse retina, BCs form ribbon synapses with their postsynaptic partners, the amacrine cells and RGCs^12,13^ (Fig. 1a). The axons of BCs, unlike those of primary sensory neurons such as the photoreceptors, have complex branching patterns and establish ribbon synapses at many locations on the dendrites of postsynaptic partners. Whether there is a stereotyped sequence in the developmental assembly of these ribbon synapses is not known.

Electron microscopy (EM) studies suggest that, unlike primary sensory neurons, ribbons in retinal BCs may be a late, rather than an early, arriving component at synapses^14–16^. This view is supported by the finding that spontaneous glutamatergic currents are detected in RGCs^17^ before ribbons are evident in the IPL^14^. These observations suggest that maturation of nascent synapses between BCs and RGCs, and their maintenance thereafter, commences after the arrival of ribbons at initial functional contact sites. To test this hypothesis directly, we performed live-cell imaging of ribbon synapses between BCs and their postsynaptic RGCs in developing and adult mouse retinas. We tracked ribbon dynamics using a new transgenic mouse line in which ribbons are tagged by a red fluorescent protein. We correlated ribbon dynamics with the appearance or maintenance of glutamatergic postsynaptic densities on RGCs to determine whether there is a strict sequence of developmental events in the assembly of bipolar-ganglion cell ribbon synapses. Specifically, we asked whether nascent bipolar-ganglion cell synapses become stabilized only after ribbons emerge at the contact sites. Finally, we analyzed synaptic development between cone BCs and the RGCs in *ribeye* knockout mice^18^ to determine whether their pattern of connectivity critically depends on the presence of ribbons.

## Results

### BCs increase their ribbon numbers rapidly before eye-opening

In the *Grm6-tdTomato* line, at least three types of ON cone BCs, Types (T) 6, 7, and 8, are labeled by expression of a red fluorescent protein (Fig. 1b, see Methods)^19^. These BC types differ in morphology, axonal and dendritic arbor size (T8 > T7 > T6) and axonal stratification depth^20–22^. These BC types differentially contact RGCs^23,24^. For example, ON-alpha RGCs receive the majority of their input from T6 bipolar cells, with some contact from T7 and T8 BCs^25,26^. Ribbons within the axon terminals of each of these BC types were revealed by immunostaining for the C-terminal binding protein 2 (CtBP2) (Fig. 1a). Digital subtraction of the immunolabel outside the volume of the axon (see *Methods*) enabled quantification of the total number of ribbons within the volume of the axon terminal. We compared the developmental time-course in the growth of the axon terminal and in the appearance of ribbons across these BC types (Fig. 1b, c). T8 BCs maintained their axonal arbor size, whereas both T6 and T7 BCs increased their axonal volume between postnatal days P9 to P21 (T6: *p* = 0.0015, T7: *p* = 0.5347, T8: *p* = 0.5347, Kruskal–Wallis rank sum test; *n* = 6–10 cells from 4–8 retinas for each age-group of each BC type; Fig. 1c). Ribbons within the axon terminals were detected by CtBP2 immunolabeling as early as P7. For all three BC types, the total number of ribbons within an axon terminal increased with age (Kruskal–Wallis rank sum test, T6: *p* = 1.102e-05, T7: *p* = 4.208e-05, T8: *p* = 0.0005), with the most rapid increase occurring before eye-opening, P15 (Fig. 1d, e). T6 and T7 BCs thus increase in their ribbon number concurrent with axonal growth, whereas T8 BCs do so without significant expansion of their axonal volume.

### Synaptic ribbons are visualized live in *RIBEYE-tagRFP* retina

**Fig. 2:**
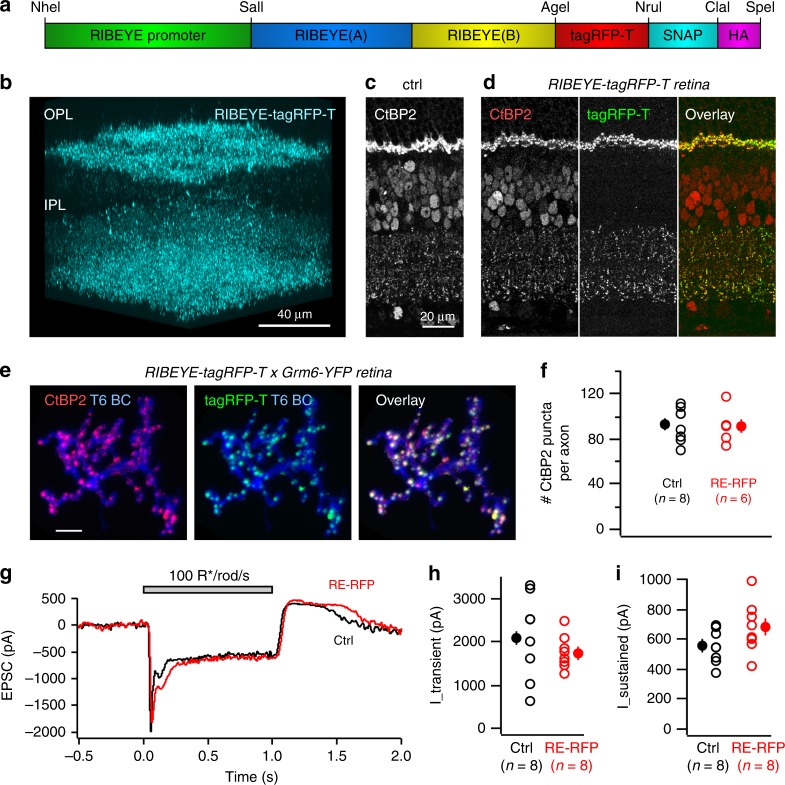
*RIBEYE-tagRFP-T* mice enable visualization of ribbons in live retina. **a** A schematic drawing of the *RIBEYE-RIBEYE:tagRFP-T* transgene. **b** Volumetric view of a two-photon reconstruction of *RIBEYE-tagRFP-T* live retina. OPL outer plexiform layer. IPL inner plexiform layer. **c** Vertical section of CtBP2 labeling in control (ctrl) retina. **d** Vertical section of CtBP2 labeling, RIBEYE-tagRFP-T (tagRFP-T), and overlay of both channels. **e** Colocalization of CtBP2 labeling and RIBEYE-tagRFP-T signal on a type 6 BC axon terminal. **f** Comparison of number of CtBP2 puncta on T6 BC axon terminal in control (ctrl; *Grm6-YFP*; eifht cells from eight retinas) and *RIBEYE-tagRFP-T* (RE-RFP) (six cells from four retinas) mice. **g** An example of light-evoked EPSC on ON-alpha RGCs in control and *RIBEYE-tagRFP-T* mice. Quantification of the amplitude of the transient (**h**) and sustained (**i**) components of the light-evoked EPSC. (**f**, **h**, and **i**: n number of cells, from three retinas for each group). P21 mice were used in all experiments. Data are presented as mean ± sem

Although observations from fixed tissue provided a clear timeline for the developmental increase in ribbon numbers within the ON BC axons, such observations do not reveal the dynamics of ribbon formation at BC synapses or enable us to ascertain whether or not ribbons are eliminated in BC axons during development. We previously created transgenic mice in which the major component of ribbons, RIBEYE, was fused with tagRFP-T (see Methods, Fig. 2a). Expression in BCs and photoreceptors is driven by the *RIBEYE* promoter; we refer to these transgenics as *RIBEYE-tagRFP-T* mice. We recently reported the use of these mice for visualizing ribbons in OFF BC axons, which are not as brightly immunolabeled by CtBP2^27^. Here, we provide a more detailed characterization of this transgenic line for its use in the current study, which requires visualization and time-lapse monitoring of ribbons in ON BCs.

In wild-type retina, punctate CtBP2 immunolabeling is found at photoreceptor axon terminals in the outer plexiform layer (OPL), as well as in BC axon terminals in the IPL (Fig. 2b, c). A similar punctate CtBP2 expression pattern was observed in *RIBEYE-tagRFP-T* mice. Moreover, tagRFP-T-positive puncta largely overlapped with CtBP2 labeling in the IPL (94.2 ± 1.3% of CtBP2 puncta colocalized with tagRFP-T puncta, and 98.5 ± 0.9% tagRFP-T puncta colocalized with CtBP2, mean ± S.E.M.; sampled from three image volumes (mean volume: 4258 μm^3^, from two retinas) of the IPL (Fig. 2d). Thus, tagRFP-T puncta faithfully represent ribbons, but a small fraction of endogenous ribbons (about 5%) may not be detected.

To determine whether tagRFP-T/CtBP2 overlap occurs within the ON BC axon terminals themselves, we crossed *RIBEYE-tagRFP-T* with *Grm6-YFP* transgenic mice, in which ON BCs are sparsely labeled by yellow fluorescent protein in addition to *RIBEYE-tagRFP-T* expression. When we isolated individual axon terminals, tagRFP-T puncta were almost always co-labeled with CtBP2 (Fig. 2e). This indicates that *RIBEYE-tagRFP-T* is a reliable marker of ribbons within BC axons. We next confirmed that there is no significant overproduction of ribbons due to overexpression of *RIBEYE-tagRFP-T*. We quantified the number of CtBP2 puncta within T6 BC axon terminals in *Grm6-YFP* (control)^28^ mice and *RIBEYE-tagRFP-T* × *Grm6-YFP* (RE-RFP) mice. In *RIBEYE-tagRFP-T* × *Grm6-YFP* retinas, the number of ribbons on a single T6 BC axon terminal was not significantly different from control retinas (Fig. 2f, control: 93.8 ± 5.3, *n* = 8 cells from 8 retinas, RE-RFP: 92.2 ± 6.0, *n* = 6 cells from 4 retinas; two-tailed Mann–Whitney test *p* = 0.948). Thus, exogenous production of the tagged RIBEYE did not alter the number of ribbons within the BCs. Finally, we confirmed that expression of tagRFP-T did not perturb synaptic transmission between BCs and RGCs in the *RIBEYE-tagRFP-T* retina. Light-evoked EPSCs were recorded from ON-alpha RGCs in both control and *RIBEYE-tagRFP-T* retinas (Fig. 2g). Previously, we showed that this RGC type receives synaptic inputs from both T6 and T7 ON BCs^25,28^. Because ribbons contribute to both transient and sustained release of vesicles, we compared both transient and sustained phases of the EPSCs, and did not observe a significant change in either current (Fig. 2h, i, *n* = 8 cells from 3 retinas for either group, two-tailed Mann–Whitney test I_sustained *p* = 0.130; I_transient *p* = 0.380). Therefore, we conclude that in *RIBEYE-tagRFP-T* mice, tagRFP-T puncta are localized at presynaptic ribbon sites, and do not disrupt synaptic transmission from the BCs.

### Ribbons are highly dynamic in immature BC axons

**Fig. 3:**
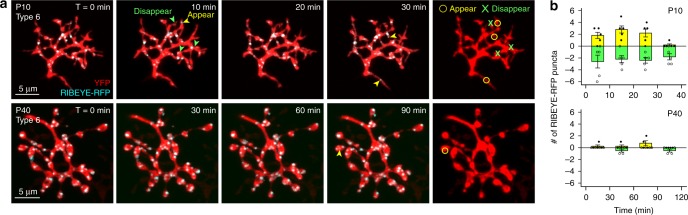
Dynamics of ribbons on axon terminals of BCs in developing and mature retinas. **a** Live imaging of ribbons within individual T6 BC axon terminals in *RIBEYE-tagRFP* × *Grm6-YFP* retinas. All RIBEYE-tagRFP-T puncta that appeared or disappeared during the imaging session were identified. Yellow arrow: punctum appeared; Green arrow: punctum disappeared. **b** Quantification of the number of RIBEYE-tagRFP-T puncta that were formed or were eliminated at P10 (five cells from three retinas) or at P40 (four cells from two retinas). Filled circle and yellow bar: puncta formed; Open circle and green bar: puncta eliminated. Data are presented as mean ± sem

To characterize the dynamic behavior of ribbon formation and elimination, we carried out live imaging of the retina from *RIBEYE-tagRFP-T* × *Grm6-YFP* double transgenics and tracked every RIBEYE-tagRFP-T punctum in individual T6 BC axons over time (Fig. 3). In both developing (P10) and mature (P40) retinas, most of the RIBEYE-tagRFP-T puncta were stable and could be identified throughout the entire imaging period (Fig. 3a). However, appearance and disappearance of RIBEYE-tagRFP-T puncta were also observed at both ages (Fig. 3a, arrowheads). At P10, the axonal processes are still very dynamic^29^. Both extensions and eliminations of processes were frequently observed. The majority of ribbons were associated with stable processes. In P10 retina, a total of 3–10 puncta appeared and 3–12 disappeared over the first 30 min of imaging (Fig. 3b top, *n* = 5 cells from 3 retinas). P10 axons contained 39.4 ± 4.8 puncta at the first time point. These changes thus represent an addition of 16.9 ± 2.2% (mean ± S.E.M.) and elimination of 17.8 ± 3.4% of the initial total ribbon number per axon over a 30 min period. In contrast, at most 1 punctum was lost or gained within a 30 min interval in the P40 retina, amongst axons that initially contained on average 93.5 ± 10.7 puncta (Fig. 3b bottom, *n* = 4 cells from 2 retinas), i.e., less than 1% change. Thus, ribbon formation and elimination are more prevalent in BC axons during development.

### Ribbons in IPL are more stable when apposed to PSD95

**Fig. 4:**
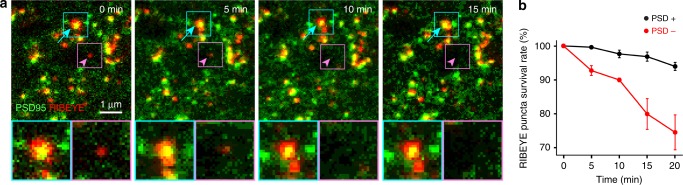
RIBEYE puncta are more stable when apposed to PSD95-FP. **a** Monitoring ribbons and postsynaptic specialization (PSD95) simultaneously in a P10 retina from *RIBEYE-tagRFP-T/PSD95-mVenus* double transgenic mice by confocal microscopy. Shown here are the maximum intensity projections of image stacks that were acquired every 5 min up to 20 min. An example of a relatively stable RIBEYE-tagRFP-T punctum apposed to PSD95-mVenus punctum (arrow and cyan box) and an unstable RIBEYE-tagRFP-T punctum not apposed to PSD95-mVenus punctum (arrowhead and magenta box). **b** Survival rate of ribbon puncta apposed (PSD95+) or not apposed (PSD95−) to PSD95-mVenus puncta (three cells from three retinas). Data are presented as mean ± sem

To determine whether the stability and instability of ribbons in the immature axons reflect their association with postsynaptic sites, we monitored pre- and postsynaptic components simultaneously by performing time-lapse imaging on *RIBEYE-tagRFP-T* × *PSD95-mVenus* mice. Previously, *PSD95-mVenus* mice have been shown to result in reliable fluorescent-labeling of endogenous PSD95 sites in the mouse brain tissues^30^. In the mouse IPL, 92.6 ± 1.0% (*n* = 3 retinal locations, one retina) of immunolabeled PSD95 puncta were fluorescently labelled in *PSD95-mVenus* mice. We acquired images every 5 min for 20 min and tracked RFP-tagged ribbons within the field of view of P10 retinas and determined whether or not each ribbon was apposed to a PSD95-mVenus punctum. We observed that RIBEYE-tagRFP-T puncta apposed to PSD95-mVenus puncta were stable throughout the imaging period (Fig. 4a arrow and cyan box). However, RIBEYE-tagRFP-T puncta that were not apposed to PSD95-mVenus gradually disappeared (Fig. 4a, arrowhead and magenta box). Quantitative comparison of the survival rate of RIBEYE-tagRFP-T puncta with or without PSD95-mVenus apposed (PSD95+ or PSD95−) confirmed that ribbons are more stable when apposed to PSD95 (Fig. 4b, *n* = 3 retinas).

### RGC PSD95 puncta are more stable when apposed to ribbons

**Fig. 5:**
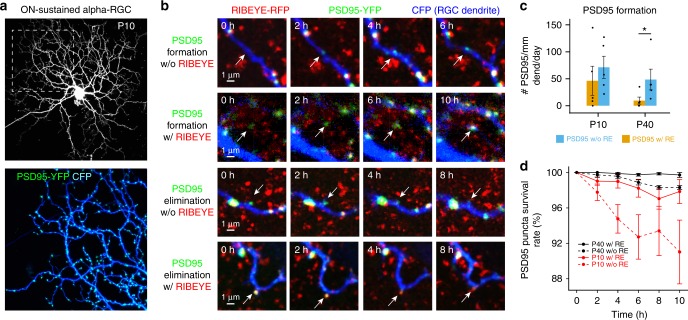
PSD95 puncta are more stable when apposed to ribbons. **a** RGCs were biolistically labeled with CFP together with PSD95-YFP. Live-cell imaging: every 2 h, up to 10 h. **b** From top to bottom: An example of PSD95 formation with (w/) or without (w/o) RIBEYE-tagRFP-T and PSD95 elimination with or without RIBEYE-tagRFP-T puncta. **c** The rate of PSD95 puncta formation with and without RIBEYE-tagRFP-T puncta at P10 and P40. **d** Survival rate of PSD95 puncta with and without RIBEYE-tagRFP-T puncta at P10 and at P40. Five live imaging experiments are performed at both P10 (five cells from four retinas) and P40 (four cells from two retinas). Data are presented as mean ± sem

We next examined directly the relationship between the stability of the PSD95-FP on the dendrites of RGCs, synaptic partners of the BCs, and their location relative to ribbons. To do so, RGCs in *RIBEYE-tagRFP-T* retinas were biolistically labelled with CFP to label their dendrites and PSD95-YFP to identify glutamatergic postsynaptic sites. Live-cell imaging was performed every 2 h up to 10 h. We focused our analysis on ON-alpha RGCs, which are identified by their characteristic dendritic morphology and stratification at both P10 (Fig. 5a) and P40. Within the time frame of imaging, four types of events were observed: PSD95 puncta formation with or without a ribbon and PSD95 puncta elimination when apposed or not apposed to a ribbon (Fig. 5b). In P10 retinas, PSD95 formation rates were similar regardless of whether or not a ribbon was present at this site. However, more PSD95 puncta were formed at sites without a ribbon in the P40 retinas (Fig. 5c; five cells from 4 P10 retinas and four cells from 2 P40 retinas, Mann–Whitney rank sum test: *p* = 0.421 for P10 and *p* = 0.036 for P40). At both ages, PSD95 puncta were, however, relatively more stable when apposed to ribbons (Fig. 5d).

### ON-alpha RGC synapse density is normal in ribbonless mice

**Fig. 6:**
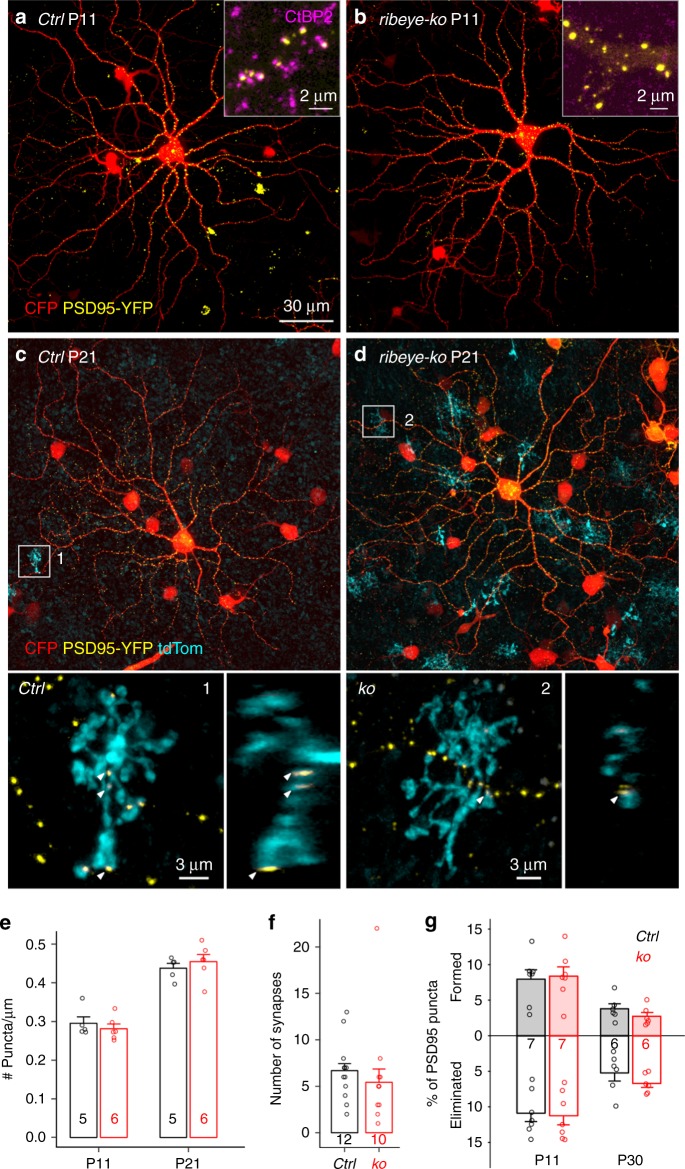
PSD95 density is unperturbed in *ribeye-ko* mice. Visualization of PSD95 puncta on dendrites of ON-alpha RGCs in littermate *control* (**a**) and *ribeye-ko* (**b**) mice. Loss of ribbon apposed to PSD95 puncta was confirmed by CtBP2 immunostaining (A and B insets). Wholemount view of a ON-alpha RGC biolistically labeled in *Grm6td-Tomato* ctrl (**c**) or *ribeye-ko* (**d**) retina at P21. Magnified views of a T6 BC synapsing onto the RGC in *ctrl* (**c**, 1) or KO (**d**, 2) retina are provided in the lower panels. Arrowheads indicate synapses between the T6 BC and the RGC. **e** Quantification of PSD95 density of ON-alpha RGCs in P11 and P21 retinas of littermate control (*ctrl*) and *ribeye-ko* (KO) mice. **f** Number of synapses between a pair of T6 BC and ON-alpha RGC. **g** Dynamics of PSD95 puncta on P11 ON-alpha RGCs during a 2 h recording period in *ctrl* and *ribeye-ko* mice. Number of cells is indicated in each bar graph. For each experiment, 2–4 retinas are used for each group. Data are presented as mean ± sem

Our time-lapse observations suggest that the presence of ribbons at contact sites increases the probability of maintenance of PSD95 at those sites. If so, we would expect a decrease in excitatory synapse density on RGC dendrites if ribbons were absent during development. To test this hypothesis, we examined the retinas of RIBEYE knockout (*ribeye-ko*) mice, which lack ribbons^18^. Surprisingly, we found that there was no reduction in PSD95 density on ON-alpha RGCs between *ribeye-ko* and littermate control retinas at both early (P11) and late (P21) developmental stages (Fig. 6a–e, two-tailed Mann–Whitney test: *p* = 0.429 for both P11 and P21). Also, synapse density between ON-alpha RGCs with their major BC partner, T6 BCs, were unchanged (Fig. 6c–f, two-tailed Mann–Whitney test: *p* = 0.147). Synapses were identified by appositions between T6 BC axonal terminals and PSD95 sites on the GC dendrites.

We then asked whether this lack of perturbation to PSD95 density on the ON-alpha RGC dendrites could be explained by alterations to the rate of BC synapse formation and elimination^19^. For instance, even if PSD95 at synaptic sites lacking ribbons were less stable, it may be that in the *ribeye-ko* mouse, PSD95 clusters form more rapidly than normal to compensate for the reduced stability. To examine this possibility, we performed live-cell imaging on ON-alpha RGCs in *ribeye-ko* and littermate control retinas at P11 and at P30. We found no significant difference in either PSD95 formation or elimination rates, for either age (Fig. 6g, *p* > 0.1 for both PSD95 formation and elimination, for either age). Thus, although the presence of synaptic ribbons is associated with a higher survival rate of apposed PSD95 puncta, it is not essential for long-term maintenance of excitatory synapses between BCs and ON-alpha RGCs.

### *Ribeye-ko* RGC light responses are altered but remain robust

**Fig. 7:**
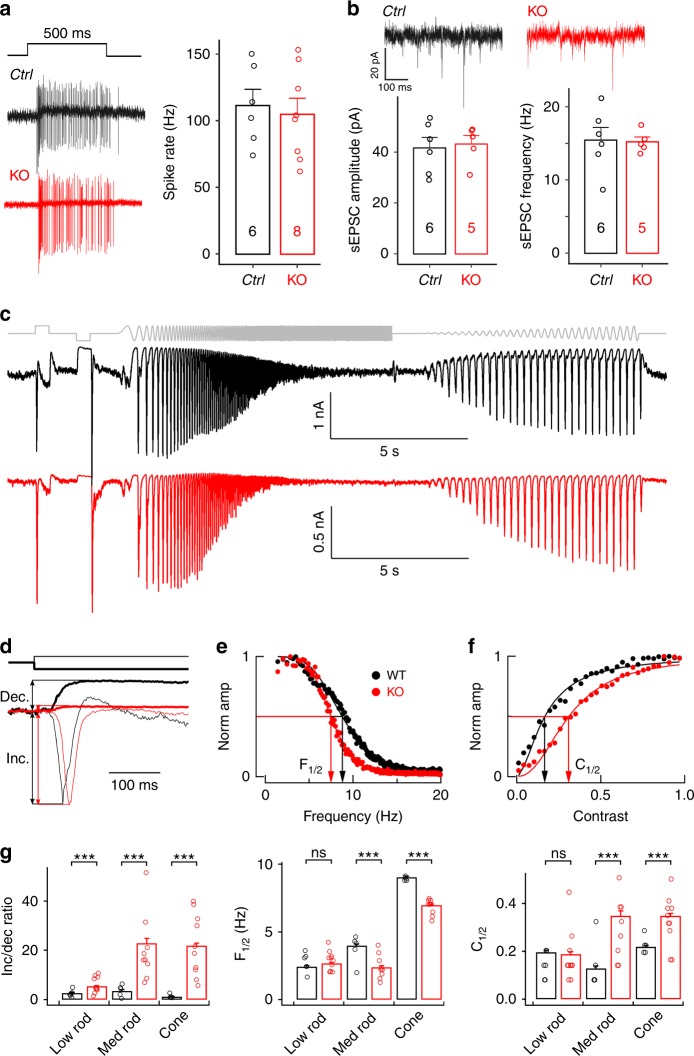
Light response properties and synaptic transmission in *ribeye-ko* mice. **a** (Left) Action potentials of ON-alpha RGCs evoked by a 500 ms light flash. (Right) Average firing rates during the stimulation duration. **b** (top) Whole-cell voltage-clamp recording of spontaneous excitatory postsynaptic currents (sEPSCs) from *ctrl* and KO. (bottom) Mean amplitude and frequency of sEPSCs. **c** Chirp stimulus (top) and example excitatory synaptic inputs to ON-alpha RGCs from ribeye knockout (red) and littermate control (black) retinas. **d** Increment and decrement responses from first section of chirp stimulus. The increment/decrement ratio determines how large the tonic excitatory input (i.e., the current suppressed by the decrement) is compared to the maximal excitatory input (i.e., the current in response to the increment). **e** Responses to frequency ramp. The amplitude of the response to each cycle of the stimulus is plotted against temporal frequency. Frequency tuning was measured from the frequency (F_1/2_) at which the response fell to half of its maximal value. **f** Responses to contrast ramp. The amplitude of the response to each cycle of the stimulus is plotted against contrast. Contrast sensitivity was measured from the contrast (C_1/2_) at which responses reached half maximum. **g** Summary of increment/decrement ratios (left), frequency tuning (middle) and contrast sensitivity (right) across cells. Low rod light level is 2 R*/rod/s, medium rod is 50 R*/rod/s and cone is 2000 R*/cone/s. *** denotes *p* < 0.001 (unpaired *t*-test). Data are presented as mean ± sem

Although the density of excitatory synapses on ON-alpha RGC dendrites was not altered, the absence of ribbons in the entire retina might be expected to disrupt synaptic transmission. To probe whether and how the absence of ribbons affects the light response of ON-alpha RGCs, we first used cell-attached recordings to record light-evoked spikes in response to a full-field light step (from darkness to 10 R*/rod/s). ON-alpha RGCs in *ribeye-ko* retina showed sustained responses similar to their wild-type counterparts (Fig. 7a). The spike rate was not significantly different from littermate controls (Fig. 7a, two-tailed Mann–Whitney test, *p* = 0.755). Maximal light-evoked excitatory inputs similarly did not differ between RGCs from *ribeye-ko* and littermate control retinas (1.0 ± 0.2 nA in 11 *ribeye-ko* RGCs vs 0.9 ± 0.2 nA in 5 control RGCs for 100% contrast steps at 2000 R*/cone/s, mean ± s.e.m.). The amplitude and frequency of spontaneous excitatory currents (sEPSC) of ON-alpha RGCs were also similar between *ribeye-ko* mice and their littermate controls (Fig. 7b, two-tailed Mann–Whitney test, amplitude *p* = 0.931, frequency *p* = 0.792). Thus, the absence of ribbons did not dramatically alter RGC responses.

To probe light responses of *ribeye-ko* RGCs in more detail, we measured excitatory synaptic inputs in response to a “chirp” stimulus consisting of three distinct components (Fig. 7c): (1) high contrast increment and decrement light steps, which probe the maximal light response and the magnitude of the tonic excitatory synaptic input; (2) a frequency ramp to measure temporal frequency sensitivity; and, (3) a contrast ramp to measure contrast sensitivity. We measured responses to these stimuli at mean light levels of 2 R*/rod/s, where rod signals cross the retina primarily through the rod bipolar pathway, 50 R*/rod/s, where rod signals cross the retina through both rod and cone bipolar pathways, and 2000 R*/cone/s, where cones dominate RGC responses^31,32^.

We used the increment and decrement responses to determine the ratio of the maximal response to the tonic excitatory input (Fig. 7d). This ratio was substantially smaller in RGCs from *ribeye-ko* retinas compared to littermate controls (Fig. 7g, left). RGC responses from *ribeye-ko* retinas also took longer to reach peak than those of controls (Fig. 7d; 88 ± 2 ms for 10 *ribeye-ko RGCs* vs 52 ± 2 ms for 5 control RGCs, mean ± sem; see Discussion). The amplitude of responses to light increments differed less than 20% between *ribeye-ko* and control RGCs; hence, the larger increment/decrement ratio in the absence of ribbons was due to smaller decrement responses. This in turn indicates less tonic excitatory synaptic input to RGCs, likely due to a lower rate of spontaneous glutamate release from bipolar cells.

We used the frequency sweep to determine the temporal frequency at which responses had fallen to half maximal (Fig. 7e). Frequency tuning differed modestly (less then two-fold) but significantly at intermediate and high light levels (Fig. 7g, middle). Finally, we used the contrast sweep to determine the contrast at which responses became half maximal (Fig. 7f). Contrast sensitivity also differed modestly but significantly at intermediate and high light levels.

These functional analyses indicate that, while RGCs continue to respond robustly in the absence of ribbons, their responses are significantly altered. More directed experiments are needed to interpret these differences mechanistically since ribbon synapses are normally present in both the photoreceptors and bipolar cells (see Discussion).

### Ribbon-free synapses are present on wild-type ON-alpha RGCs

**Fig. 8:**
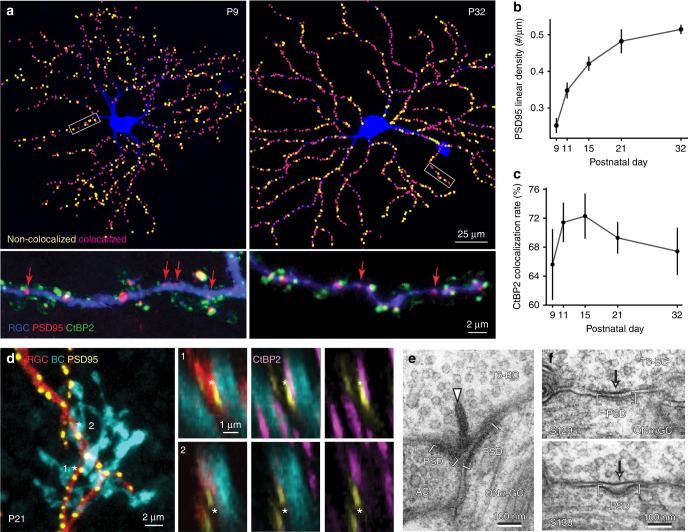
BCs form ribbon-containing and ribbon-free contacts with wild-type RGCs. **a** (Top panels) Relationship of PSD95-YFP puncta on ON-alpha RGCs (blue) with ribbons (CtBP2 labeling) at P9 and P32. Contact sites with PSD95-YFP and CtBP2 apposed are indicated as ‘colocalized’ (magenta) whereas PSD95-YFP sites without CtBP2 are marked as ‘non-colocalized’ (yellow). (Bottom panels) Magnified views of the stretch of dendrite within the rectangles. Red arrows indicate PSDS95 puncta not apposed to CtBP2 puncta. **b** Developmental increase of PSD95 density on the dendrites of ON-alpha RGCs. **c** Percentage of CtBP2 puncta apposed to PSD95 puncta on the dendrites of ON-alpha RGCs across development. *N* = 4–6 cells from 3–6 retinas. **d** Maximum intensity projections of confocal image stacks of *en face* views of T6 BC axon terminals and the dendrites and PSD95 puncta of an ON-alpha RGC. Two example synaptic contacts are marked with the asterisks. Magnified views for both contacts are shown on the right. (1) ribbon synaptic contact; (2) ribbonless synaptic contact. **e** Electron micrograph showing a typical ribbon (arrowhead) synapse between a T6 BC axon and a dyad of ON-alpha RGC and amacrine cell (AC) dendrites. Both GC and amacrine cell membranes are line with PSDs (brackets). **f** Two consecutive electron micrographs (S129 and S130) showing a non-ribbon contact (arrows) for the same T6 BC and ON-alpha GC pair shown in **e**. The GC membrane is slightly concave and clearly lined by a postsynaptic density (brackets). Data are presented as mean ± sem

The ability to form stable ribbon-free synapses between BCs and RGCs in *ribeye-ko* mice raises the question of whether or not BCs normally form ribbon-free synapses with RGCs. Indeed, recent ultrastructural observations in the mouse retina suggest that some BCs make synapses that lack ribbons^22^. But, the prevalence of such synapses and whether they represent connections made between specific synaptic partners are unknown. We thus immunostained PSD95-FP transfected RGCs with CtBP2 at different ages between P9 and P32 (examples, Fig. 8a), quantified the linear density of PSD95 on ON-alpha RGCs across these ages (Fig. 8b), and obtained the percentage of PSD95 sites on these RGCs that were not apposed to ribbons (Fig. 8c). Although ON-alpha PSD95 puncta density increased with age (Kruskal–Wallis rank sum test: *p* = 0.0008), not all PSD95-FP sites were apposed to CtBP2 puncta during development (Fig. 8c). However, a similar proportion (about 30%) of PSD95-FP puncta remained ribbon-free even at P32 despite the overall increase in PSD95 density (Fig. 8c, Kruskal–Wallis rank sum test, *p* = 0.46, *n* = 4–6 cells from 3–6 retinas for each age-group), suggesting that both ribbon-containing and ribbon-free synapses increased with maturation.

Finally, we asked whether or not the ribbon-free contacts were made with a specific BC type. We thus examined the synapses formed between pairs of T6 BCs and ON-alpha RGCs using confocal microscopy, and found that an individual T6 axon terminal can form ribbon-containing and ribbon-free synapses (Fig. 8d) with the ganglion cell. In 5 out of 12 T6 BC-RGC pairs, we observed one or two PSD95 puncta apposed to axon terminals without a CtBP2 puncta. On average, 6.8 ± 3.1% of the synapses between T6 BC and ON-alpha RGC lack ribbons. The ribbon-free PSD95-FP puncta are likely at synapses, rather than due to an overexpression of PSD95. This is because a recent EM study showed that BCs make ribbon-free contacts with RGCs in mouse retina^22^. We extended these EM findings by further analysis of serial transmission EM sections, and provide examples here of ribbon containing and ribbon-free synaptic contacts between morphologically identified T6 BCs and an ON-alpha RGC (Fig. 8e, f). Ribbon-free contacts were identified by the presence and apposition of pre- and postsynaptic densities at the BC-RGC contact site (Fig. 8f). Thus, a BC type or even an individual BC can make both ribbon containing and ribbon-free synapses with RGCs; ribbon-free synapses are thus not a transient developmental anomaly.

## Discussion

We used transgenic mice, live-cell imaging, and quantitative approaches to track the dynamics of ribbons during synaptogenesis and maturation of the mouse inner retina. We adopted these approaches together with *ribeye-ko* mice to determine whether ribbons are necessary for stabilizing nascent contacts between retinal BCs and RGCs. Our experiments provide a dynamic view of the assembly of cone bipolar-ganglion cell ribbon synapses, and also demonstrate clear but surprisingly modest effects on the connectivity and RGC signaling in both rod and cone retinal circuits in the absence of ribbons throughout the retina.

A previous EM study showed that synaptic ribbons appear in the mouse retinal IPL around P10, and increase in number until about 3 weeks after birth^14^. Our present study showed that, during this period, the number of ribbons within individual T6, T7, or T8 BC axon terminals increased steadily, but at different rates. The number of ribbons within individual T6 and T7 BC terminals at P21 identified under light microscopy were comparable to previous measurements obtained from EM reconstructions of these BC types in 9-week-old adults^22^. Thus, T6 and T7 BCs appear to reach their adult ribbon numbers at juvenile ages. EM reconstruction of a T8 BC uncovered fewer ribbons in this BC type in the adult retina compared to our analysis of T8 BCs at P21^22^. This may be because the EM reconstructed T8 BC is smaller than the average size of the cells we analyzed here. Another possibility is that after P21 T8 BCs undergo axonal pruning and ribbon synapse elimination, processes known to occur at their dendrites in the outer retina^21^.

Analysis of fixed tissue cannot reveal whether ribbons are always maintained once localized to a synapse. The *RIBEYE-tagRFP-T* transgenic mouse enabled us to monitor ribbon dynamics in individual BC axon terminals. We observed both the formation and elimination of ribbons in immature and mature retinas. Although both additions and eliminations were captured during our time-lapse imaging, we did not observe a net increase in ‘synapses’ at the end, suggesting that the overall increase of ribbon number with age occurs gradually over hours to days, consistent with the estimation by Fisher (1979)^14^. Not surprisingly, the frequency of both additions and eliminations declined with age. The relatively few additions and eliminations of ribeye puncta in mature BCs occurred at stable axonal boutons. Whether these events constitute changes in the organization or distribution of active zones or even formation or loss of specific synapses remains to be elucidated. Regardless, although rare, such changes suggest that BC axons retain ongoing plasticity in the adult retina.

Our time-lapse analysis of ribbon synapse development differs from past work on synaptogenesis of cultured neurons in several ways. Previous time-lapse analyses tracked synaptogenesis by marking presynaptic terminals by expression of fluorescently-tagged synaptic vesicle proteins^33–36^ or bassoon^37^, or by FM-dye update^37^, and postsynaptic differentiation by PSD95-GFP expression^33–35,37,38^ or by glutamate receptor accumulation^37,39^. While findings in culture suggest that presynaptic proteins generally appear at the site of contact a few hours before PSD95 accumulation^35,37^, we find that ribbons could appear after a PSD95 punctum is already at the contact site. This may not be surprising because EM observations suggest that ribbons are likely localized after synaptic vesicles are already present at BC contacts with RGCs. Further, although presynaptic components have been found to transport as ‘packages’ to synaptic sites^38^, we did not observe transport of ribbons along the BC axon shaft or within its branches. At both P11 and P40, ribeye puncta emerged over time at sites of contacts between BCs and RGCs, presumably upon localized accumulation of this protein. Ribeye puncta were largely added to stable parts of the axonal terminal, rather than at filopodia. It remains possible, however, that ribbon precursors such as those previously identified in photoreceptors^40^ are also present in immature BC axons, and that their small size precludes visualization by fluorescence imaging under confocal microscopy. Ribeye puncta in the BC axons can appear very rapidly, within tens of minutes. Fast accumulation of ribeye proteins has also been shown in zebrafish skin cells by measuring fluorescence recovery after photobleaching^41^.

Previous culture studies of hippocampal neurons demonstrate that synapse elimination can occur after pre- and postsynaptic components are assembled at a contact site^33–35,37^. Likewise, we found that ribeye puncta that are apposed to PSD95 puncta can be eliminated, suggesting that loss of ribeye at least at some locations is likely associated with removal at synapses. Ribeye puncta that are apposed to PSD95 are, however, relatively more stable compared to those that are not associated with PSD95 at the time of imaging. Some ribbons that were not apposed to PSD95 may appear stable because they may be at glutamatergic sites containing other PSDs, such as SAP102^42,43^. If so, the survival rates for ribbons at nascent synapses lagging in postsynaptic maturation would be even lower. We also observed that PSD95 puncta that were not apposed to ribbons are less stable. The loss of these postsynaptic puncta may be due to a failure of the presynaptic sites to further differentiate, or these may be PSD95 sites previously associated with a ribbon that had dismantled. Indeed, we have on occasion observed the disassembly of PSD95 clusters at contacts that contain both these structures. Previous work suggests that loss of either pre or postsynaptic components can lead to synapse disassembly^44^, but whether loss of ribeye or PSD95 puncta during our imaging period reflects the eventual loss of physical contact remains to be confirmed. This can be achieved in the future by combining time-lapse imaging of ribbons and PSD95 with serial EM reconstructions, using correlative fluorescence imaging and serial block-face scanning EM methods^45^.

Although our time-lapse analysis suggests that nascent BC-RGC synapses are more stable when ribbons are present, such observations cannot predict how BC-RGC synaptic development would be affected without ribbons over an extended time-scale of weeks in vivo. We found that ON-alpha RGCs in *ribeye-ko* mice possessed normal PSD95 puncta densities and continued to respond robustly to light stimuli. Quantitative analysis, however, revealed alterations in light responses of RGCs from *ribeye-ko* retinas compared to littermate controls, with decreases in spontaneous release, frequency sensitivity and contrast sensitivity. Light-evoked responses, however, were overall surprisingly normal. This was unexpected not only because of the complete loss of ribbons in the IPL, but also because of their absence in the photoreceptor terminals.

Previous work showed that fast and sustained transmission from rod BCs to AII amacrine cells were reduced ~4-fold in the *ribeye-ko*, although rod BCs still formed synaptic dyads^18^. This is substantially larger than the <2-fold changes we observe in the light response, particularly at low light levels in which responses traverse the retina through the rod bipolar pathway. Comparison of our current findings with the effects on rod BC circuits^18^ thus leads to two conclusions: (1) ribbons are not critical for the structural formation of synapses between BCs and RGCs, and (2) direct measures of synaptic function^18^ can reveal deficits that have modest effect on light-evoked responses. Indeed, marked reductions in both sustained and phasic release have been observed after acute photodamage of ribbons in both cones and bipolar cells^46^. Further work will be required to determine whether the relatively small impact of loss of ribbons on light-evoked responses reflects redundant synaptic mechanisms or compensatory changes when ribbons are absent.

Like the ON-alpha RGC, stimulus-evoked spike rates of afferent neurons postsynaptic to lateral line hair cells in zebrafish *rib*eye mutants appear normal, although there is increased exocytosis^47^. Our recordings from the RGCs and the zebrafish work contrast with recent findings showing impaired temporal precision in sound encoding involving inner hair cells of *ribeye-ko* mice^48^. Thus, different circuits even within a sensory modality (rod vs cone BCs; hair cells in fish and mice) may compensate differentially for loss of ribbons.

The ability of cone BCs to form conventional (ribbon-free) synapses with RGCs may seem abnormal in *ribeye-ko* mice. However, conventional synapses between BCs and RGCs have been documented previously in goldfish, salamander, rabbit, mouse, and primate retinas^22,49–52^. We also show here the presence of ribbon-free synapses on adult ON-alpha RGCs in wild-type retina. These synapses could contribute to the modest effects of removal of ribbons on RGC light responses. A parsimonious explanation for two types of BC synapses is that ribbon-free synapses on the RGC dendrites are formed largely with a specific BC type. Although this remains plausible, we found that an individual T6 BC cell makes both ribbon and ribbon-free synapses with ON-alpha RGCs. Future experiments are needed to ascertain whether non-ribbon bearing BC synapses represent functional divergence at the level of individual synapses between a BC and RGC pair. However, the inconsistent proportion of ribbon-free synapses across axons of a specific BC type (T6 here) suggests that ribbon-free synapses are unlikely to have a unique function at least in T6-ON-alpha RGC circuits. It would be still be informative in the future to determine whether other RGC types receive input at ribbon-free synapses, and if so, whether connections with specific BC input types are involved.

The presence of ribbon-free synapses in wild-type adult retina and the normal cone BC synapse density on the ON-alpha RGCs in the *ribeye-KO* raises the question of what presynaptic proteins may be engaged in stabilizing these BC synapses in wild-type and *ribeye-KO* retinas. Disruption of several pre- or postsynaptic molecules in the outer retina that provide a trans-synaptic link between photoreceptors and BCs have been identified (reviewed by ref. ^53^). Of these, loss of CaV_1.4_, specifically the α2δ-4 auxiliary subunit of this voltage-gated calcium channel, results in the disappearance of full-length ribbons at rod photoreceptor terminals; rod and also cone postsynaptic arrangements are disrupted^54,55^. The α2δ-2 subunit has similarly been found to be important for organizing synapses of inner hair cells in mice^56^. The identity and molecular organization of the calcium channels localized to presynaptic terminals of cone BCs have not yet been fully defined. But, future work assessing the cone BC output synapses in mouse mutants with BCs lacking various voltage-gated calcium channels will be informative, potentially identifying a role for such channels in regulating cone BC-RGC synaptic development.

In summary, our experiments with the *RIBEYE-tagRFP-T* mice demonstrate that although synaptic ribbons can influence the stability of nascent BC synapses in the inner retina, their presence is not essential for establishing wild-type synapse densities between a type of cone BC and its target RGC in vivo. This finding is in line with the initial characterization of the *ribeye-ko* mouse^18^, which demonstrated a largely normal ultrastructure of retinal ribbon synapses, except for the complete absence of synaptic ribbons, and normal synaptic architecture of both retinal photoreceptor synapses and rod BC synapses. Similarly, RGCs exhibited surprisingly modest alterations in their light responses in the absence of ribbons. Because excitatory synapse densities on ON-alpha RGCs appear unperturbed, future studies can exploit the circuitry of this RGC type to determine whether ribbons are dispensable for the function of some cone BCs and not others, for example those providing major versus minor input onto the RGC.

## Methods

### Mice

All experiments were conducted following animal protocols approved by the Institutional Animal Care and Use Committee at University of Washington. All procedures in these protocols are in compliance with the National Institute of Health Guidelines for the Care and Use of Laboratory Animals. Mice ranging in age between postnatal days (P) 9–60, of either sex were used. *RIBEYE-ribeye-tagRFP-T* mice were generated to visualize ribbons in the retina (see details below). *PSD95-CreNABLED (PSD95-mVenus)* mice were used to identity sites of excitatory postsynaptic densities (PSD95) in the retina^30^. PSD95-CreENABLED mice were derived upon crossing PSD95-ENABLED mice with germline HPRT-Cre mice (stock NO: 004302, Jackson Labs). BCs were visualized either using *Grm6-tdTomato* mice^19,28^ or *Grm6-YFP-STOP-TeNT* mice^57^, which expresses YFP but not tetanus toxin, TeNT (we refer to these mice as *Grm6-YFP*). In both these transgenic lines, rod BCs and cone BC Types 6, 7, and 8 are sparsely labeled^21,28^. These cone BC types were distinguished by their axonal morphology and size, and by their axonal stratification levels in the IPL^12,22^ (see BC identification section). RIBEYE knockout mice were bred from heterozygous breeding pairs and littermate wild-type mice were used as controls^18^.

The transgenic RIBEYE vector was cloned into a pEGFP-N1 based vector backbone with a modified multiple cloning site (MCS) containing BssHII, SacI, SacII, NheI, SalI, AgeI, NruI, ClaI, SpeI, BamHI, and NotI restriction sites using standard molecular biology methods. The ≈5.1 kb RIBEYE mouse promoter was obtained from a mouse BAC clone (RP23-211A9; AC119806 (from mus musculus chromosome 7) and cloned into the NheI/SalI sites. Full-length rat RIBEYE cDNA was cloned into the SalI/AgeI sites of the MCS. At the carboxyterminus of RIBEYE, we cloned in frame three tags, i.e., tagRFP-T^58^, SNAP2.1-tag^59^, and an hemagglutinin (HA)-tag. TagRFP-T was cloned into the AgeI/NruI sites; SNAP2.1-tag into the NruI/ClaI site and the HA-tag into the ClaI/SpeI sites of the modified MCS. All cloning steps were verified by sequencing. The transgenic vector contained a hGH polyadenylation signal^60^ placed between the BamHI and NotI site of the transgenic vector. The transgenic construct was excised via the flanking BssHII and NotI sites and gel-purified prior to pronucleus injection, which was performed by Frank Zimmermann/Sascha Dlugosz (IBF; University of Heidelberg). Positive founder animals were identified via PCR using the following primer pairs: F1(GCTTCGAATTCGGCACGAGGA), R1(CCTGGTGCCCCTGGATGGGT); F2(CCACGGAGATCCGCCGAGCA), R2 (CGCCCTCGGATGTGCACTTGA); F3(GCTCGCCGTGAAAGAGTGGC) R3 (GTCCGGGAGCCTGGGGAGAAA).

### Tissue preparation

For immunohistochemistry, biolistic transfection, and live-cell imaging, mice were deeply euthanized with isoflurane, decapitated, and enucleated. Retinas were then dissected in oxygenated mouse artificial cerebrospinal fluid (mACSF, pH 7.4) containing (in mM) 119 NaCl, 2.5 KCl, 2.5 CaCl_2_, 1.3 MgCl_2_, 1 NaH_2_PO_4_, 11 glucose, and 20 HEPES at room temperature. The retina was isolated from the eyecup first and mounted on a nitrocellulose membrane disc (Millipore) with retinal ganglion cell side up. For electrophysiology, mice were dark-adapted overnight and sacrificed by cervical dislocation under infrared illumination. The retina was mounted photoreceptor side down on poly-lysine coated cover slides (BD Biosciences) and perfused with oxygenated Ames solution (Sigma) heated at 32 °C.

### Immunohistochemistry

The retinas were fixed for 15 min in 4% paraformaldehyde in mACSF. The retinas were first incubated in blocking solutions containing 5% normal donkey serum for 2 h under room temperature. Then, they were incubated in primary antibodies for three nights and secondary antibodies overnight at 4^o^C. Primary antibodies used in this study were: CtBP2 (1:1000, mouse, BD Biosciences), PSD95 (1:500, mouse, Abcam), and Lucifer Yellow (1:1000, rabbit, Invitrogen). Secondary antibodies were anti-isotypic DyLight (1:1000, Jackson ImmunoResearch) or Alexa conjugates (1:1000, Invitrogen).

### Biolistic transfection

Gold particles (1.6 μm diameter, 12.5 mg, Bio-Rad) were coated with DNA plasmids (24 μg) encoding cerulean or tdTomato, and PSD95-YFP or PSD95-CFP (12 μg) under the control of the cytomegalovirus (CMV) promoter. The particles were delivered to RGCs using a Helios gene gun (40 psi, Bio-Rad). Biolistically transfected retinas were kept in mACSF in a humidified, oxygenated chamber at 33 °C for 22–24 h.

### BC identification

Identification of types of ON BCs was mainly relied on the size and the morphology of dendritic arbors and axon terminals, together with the level of axonal stratification within IPL in order to distinguish Type 6, 7, 8, and RBCs^21^. CtBP2 immunolabeling and RIBEYE-tagRFP-T helped the determination of the axonal stratification as they label ribbons throughout IPL. The following are the features that are helpful in distinguishing different ON BC subtypes^12,20–22^. Type 6 BCs have axon terminals whose branches stratify widely through S3 to S4. Their claw-like dendritic terminals are clustered into a few locations in OPL. Typically, the dendritic and axon terminal areas of Type 6 BCs are larger than those of RBCs but smaller than those of Type 7 and Type 8 BCs. Type 7 BCs are distinguished by the axon terminals that are narrowly stratified at a layer slightly shallower (i.e., closer to INL) than those of Type 6 and Type 8 BCs. Type 8 BCs are characterized by their sparse and widespread dendritic arbors and axonal branches. RBCs have the smallest dendritic and axonal terminal areas among ON BCs with its large lobular axonal boutons that reach the deepest in IPL (i.e. closest to ganglion cell layer).

### Image acquisition

For the live-cell imaging of BCs and RGCs, retinas were placed in a chamber and superfused with oxygenated mACSF at 2–3 ml/min and heated at 33–34 °C. The images were acquired using either an Olympus FV1000 confocal microscope and a 60x water immersion objective (NA 1.1), or using a Leica TCS SP8 laser scanning confocal microscope with a 63x water immersion objective (NA 1.2). Voxel sizes were 0.1 × 0.1 × 0.3 μm. For fixed tissue, images stacks were acquired using the Olympus FV1000 with a 60x oil immersion objective (NA 1.35) or with the Leica TCS SP8 with a 63x oil immersion objective (NA 1.4), and a voxel size of 0.1 × 0.1 × 0.3 μm for imaging the entire retinal ganglion cell, or 0.05 × 0.05 × 0.25 μm for imaging individual BCs.

### Image analysis

All the raw images acquired were median-filtered and converted to 8 bit using ImageJ before further processing. For live-cell imaging, time-lapse image stacks were aligned using Amira (Thermo-Fisher Scientific). Parameters required to align the images were entered into a custom-written Matlab (MathWorks) script to trim the margin of the images, which enabled alignment of the image stack of the time series.

For counting or tracing ribbons on individual BC terminals, a binary mask was created using the Labelfield function in Amira (Thermo-Fisher Scientific) software. The CtBP2 or RIBEYE-tagRFP-T signals within the individual axon terminals were isolated by multiplying original images by the mask using the Arithmetic function in Amira.

For counting or tracking PSD95 puncta on the dendrites of individual RGCs, skeletonization of the dendrites was first performed using Imaris (Bitplane). Manual corrections were made after applying the automatic filament tracing function. An enlarged mask around the dendrites was created and applied on PSD95 image stacks.

The puncta were identified using a custom-written Matlab (The Mathworks Inc) file and Imaris (Bitplane)^57^. Manual corrections were made whenever necessary. Puncta that were present over time were connected using Tracks function. The dot information was saved and exported. The formation and elimination rate as well as the survival rate were calculated using a custom-written Matlab script.

RIBEYE-tagRFP-T puncta and PSD95-FP puncta were visually evaluated in 3D and colocalized puncta were identified manually using Imaris. This information was exported from Imaris as a Matlab readable file. The formation and elimination rate and the survival rate of RIBEYE-tagRFP-T puncta and PSD95-FP puncta were then analyzed in relation to their colocalization status using a custom-written Matlab script.

### Electron microscopy

T6 BCs and an ON-alpha RGC were reconstructed from the same series of transmission EM images photographed at a magnification of ×3000 as in a previous study^22^. Ribbon containing and ribbon-free synaptic contacts were first chosen on print paper at ×12,000 after four-fold enlargement. Next, they were re-photographed at ×20,000 with section tilting between - 25 and + 25 degrees.

### Electrophysiology

Cell-attached and whole-cell voltage-clamp recordings were made from on ON-alpha RGCs in wholemount retinas. Light stimuli were delivered from a calibrated 470 nm LED focused on the photoreceptors through the microscope condenser. ON-alpha RGCs were targeted based on somal size and their characteristic spike response to a light step. Whole-cell voltage-clamp recordings were used to isolate excitatory synaptic inputs by clamping at the reversal potential for inhibition (~-60 mV), which was experimentally determined for each cell^25,61^. Spontaneous excitatory synaptic currents (sEPSCs) were measured while superfusing the retina with L-(+)-2-Amino-4-phosphonobutyric acid (L-AP4, 10 μM, Tocris Bioscience) to hyperpolarize On bipolar cells and reduce the EPSC rate. Series resistance (6–8 MOhm) was 70% compensated in whole-cell recordings.

### Statistics

All statistics were performed using R version 3.4.1 (R Foundation for Statistical Computing; https://www.R-project.org). Where the number of samples did not enable us to test for normality, linearity or homoscedasticity of the data, we used a two-tailed Mann–Whitney test to compare two groups of data. Significance was determined at *p* < 0.05. Data are presented as mean ± sem, unless noted otherwise. To determine whether a trend (e.g., increasing axon size with age) was significant, we used the Kruskal–Wallis rank sum test.

### Reporting summary

Further information on research design is available in the Nature Research Reporting Summary linked to this article.

## Supplementary information

Reporting Summary

## Data Availability

The dataset generated and analyzed in this study are available upon request to the corresponding author.

## Code availability

The dataset generated and analyzed in this study are available upon request to the corresponding author.
