# Supplementary material for: Dynamic assembly of ribbon synapses and circuit maintenance in a vertebrate sensory system
Source: Nat Commun. 2019 May 15;10:2167. doi: 10.1038/s41467-019-10123-1 (PMC6520400; doi:10.1038/s41467-019-10123-1)
Supplement: Supplementary file 1 — Reporting Summary [file 41467_2019_10123_MOESM1_ESM.pdf]

nature research

Corresponding author(s): *Double-blind peer review submissions: write DBPR and your manuscript number here instead of author names.*

Reporting Summary

Nature Research wishes to improve the reproducibility of the work that we publish. This form provides structure for consistency and transparency in reporting. For further information on Nature Research policies, see Authors & Referees and the Editorial Policy Checklist.

Statistical parameters

When statistical analyses are reported, confirm that the following items are present in the relevant location (e.g. figure legend, table legend, main text, or Methods section).

n/a	Confirmed	
☐	☒	The exact sample size (*n*) for each experimental group/condition, given as a discrete number and unit of measurement
☐	☒	An indication of whether measurements were taken from distinct samples or whether the same sample was measured repeatedly
☐	☒	The statistical test(s) used AND whether they are one- or two-sided<br>*Only common tests should be described solely by name; describe more complex techniques in the Methods section.*
☒	☐	A description of all covariates tested
☒	☐	A description of any assumptions or corrections, such as tests of normality and adjustment for multiple comparisons
☐	☒	A full description of the statistics including central tendency (e.g. means) or other basic estimates (e.g. regression coefficient) AND variation (e.g. standard deviation) or associated estimates of uncertainty (e.g. confidence intervals)
☐	☒	For null hypothesis testing, the test statistic (e.g. *F*, *t*, *r*) with confidence intervals, effect sizes, degrees of freedom and *P* value noted<br>*Give P values as exact values whenever suitable.*
☒	☐	For Bayesian analysis, information on the choice of priors and Markov chain Monte Carlo settings
☒	☐	For hierarchical and complex designs, identification of the appropriate level for tests and full reporting of outcomes
☒	☐	Estimates of effect sizes (e.g. Cohen's *d*, Pearson's *r*), indicating how they were calculated
☐	☒	Clearly defined error bars<br>*State explicitly what error bars represent (e.g. SD, SE, CI)*

Our web collection on statistics for biologists may be useful.

Software and code

Policy information about availability of computer code

Data collection	ephys data was collected by Symphony Data Acquisition System http://www.open-ephys.org/symphony
Data analysis	ImageJ, Imaris, Amira, Custom Matlab and R codes

For manuscripts utilizing custom algorithms or software that are central to the research but not yet described in published literature, software must be made available to editors/reviewers upon request. We strongly encourage code deposition in a community repository (e.g. GitHub). See the Nature Research guidelines for submitting code & software for further information.

Data

Policy information about availability of data

All manuscripts must include a data availability statement. This statement should provide the following information, where applicable:

- Accession codes, unique identifiers, or web links for publicly available datasets
- A list of figures that have associated raw data
- A description of any restrictions on data availability

The dataset generated and analyzed in this study are available upon request to the corresponding author.

Field-specific reporting

Please select the best fit for your research. If you are not sure, read the appropriate sections before making your selection.

☒ Life sciences ☐ Behavioural & social sciences ☐ Ecological, evolutionary & environmental sciences

For a reference copy of the document with all sections, see nature.com/authors/policies/ReportingSummary-flat.pdf

Life sciences study design

All studies must disclose on these points even when the disclosure is negative.

Sample size	*Describe how sample size was determined, detailing any statistical methods used to predetermine sample size OR if no sample-size calculation was performed, describe how sample sizes were chosen and provide a rationale for why these sample sizes are sufficient.*
Data exclusions	Image stacks with high quality (high signal-to-noise ratio) were analyzed. All analyzed data were included in the paper.
Replication	*Describe the measures taken to verify the reproducibility of the experimental findings. If all attempts at replication were successful, confirm this OR if there are any findings that were not replicated or cannot be reproduced, note this and describe why.*
Randomization	We allocated samples into experimental groups by their age or genotypes. The mice within each group were randomly chosen.
Blinding	Most of the experiments were carried out by one investigator at a time and this person has to track the genotype/age of the sample collected. The investigator conducted electro physiological experiments and confirmed the sample group after the recording.

Reporting for specific materials, systems and methods

Materials & experimental systems

n/a	Involved in the study
☐	☒ Unique biological materials
☐	☒ Antibodies
☒	☐ Eukaryotic cell lines
☒	☐ Palaeontology
☐	☒ Animals and other organisms
☒	☐ Human research participants

Methods

n/a	Involved in the study
☒	☐ ChIP-seq
☒	☐ Flow cytometry
☒	☐ MRI-based neuroimaging

Unique biological materials

Policy information about availability of materials

Obtaining unique materials	All unique materials are available upon request from the authors.

Antibodies

Antibodies used	Primary antibodies used in this study were: CtBP2 (1:1000, mouse, BD Biosciences), PSD95 (1:500, mouse, Abcam) and Lucifer Yellow (1:1000, rabbit, Invitrogen). Secondary antibodies were anti-isotypic DyLight (1:1000, Jackson ImmunoResearch) or Alexa conjugates (1:1000, Invitrogen).
Validation	References:<br>CtBP2: Morgan, J. L., Schubert, T., & Wong, R. O. (2008). Developmental patterning of glutamatergic synapses onto retinal ganglion cells. Neural development, 3(1), 8.<br><br>PSD95: Oh, E. C., Khan, N., Novelli, E., Khanna, H., Strettoi, E., & Swaroop, A. (2007). Transformation of cone precursors to functional rod photoreceptors by bZIP transcription factor NRL. Proceedings of the National Academy of Sciences, 104(5), 1679-1684.<br><br>Lucifer Yellow: Bleckert, A., Schwartz, G. W., Turner, M. H., Rieke, F., & Wong, R. O. (2014). Visual space is represented by nonmatching topographies of distinct mouse retinal ganglion cell types. Current Biology, 24(3), 310-315.

Animals and other organisms

Policy information about studies involving animals; ARRIVE guidelines recommended for reporting animal research

Laboratory animals	Mice ranging in age between postnatal days (P) 9-60, of either sex were used. Strains included: C57BL/6J, PSD95-CreNABLED, RIBEYE-ribeye-tagRFP-T, Grm6-tdTomato, Grm6-YFP-STOP-TeNT, Ribeye-ko
Wild animals	The study did not involve wild animals.
Field-collected samples	The study did not involve field-collected samples.
